# *Helicobacter pylori *in apparently healthy children aged 0-12 years in urban Kampala, Uganda: a community-based cross sectional survey

**DOI:** 10.1186/1471-230X-10-62

**Published:** 2010-06-16

**Authors:** Elin Hestvik, Thorkild Tylleskar, Deogratias H Kaddu-Mulindwa, Grace Ndeezi, Lena Grahnquist, Edda Olafsdottir, James K Tumwine

**Affiliations:** 1 Centre for International Health, University of Bergen, Årstadveien 21, N-5009 Bergen, Norway; 2 Department of Paediatrics, Haukeland University Hospital, N-5021 Bergen, Norway; 3 Department of Microbiology, Makerere University Medical School, P.O Box 7072, Kampala, Uganda; 4 Department of Paediatrics and Child Health, Makerere University Medical School, P.O Box 7072, Kampala, Uganda; 5 Department of Women's and Children's Health, Karolinska Institutet, 17176 Stockholm, Sweden

## Abstract

**Background:**

*Helicobacter pylori *is one of the most common causes of bacterial infection in human beings. Studies have showed a high prevalence of *Helicobacter pylori *among people in low-income countries and colonization early in life. A monoclonal antigen test, performed on faeces, HpSA^®^ImmunoCardSTAT, has a high sensitivity, specificity and accuracy and the faecal test can be performed in all ages, also in resource-limited settings. The main objective of this study was to determine the prevalence and factors associated with *Helicobacter pylori *colonization in apparently healthy children aged 0-12 years in urban Kampala, Uganda.

**Method:**

We tested 427 apparently healthy children, age 0-12 years (211 males, 216 females), in a cross sectional survey for *Helicobacter pylori *colonization using HpSA ^®^ImmunoCardSTAT. A short standardized interview with socio-demographic information and medical history was used to assess risk factors.

**Results:**

The overall prevalence of *Helicobacter pylori *in the 427 children was 44.3% (189 out of 427). Early colonization was common, 28.7%, in children younger than 1 year of age. The age specific rates were 46.0% in children age 1- < 3 years, 51.7% in children age 3- < 6 years, 54.8% in children age 6- < 9 years and 40.0% in children age 9- < 12 years. There was a significant difference in prevalence by gender; female 38.5% versus male 49.8% and by type of housing; permanent house 38.5% versus semi-permanent house 48.6%. Congestive living and education level of the female caretaker showed a clear trend for a difference in prevalence. Factors independently associated with *Helicobacter pylori *colonization included: drugs taken last three months, using a pit latrine, sources of drinking water and wealth index.

**Conclusion:**

The prevalence of *Helicobacter *pylori colonization among urban Ugandan children is high at an early age and increases with age. The impact of Helicobacter pylori colonization on children's health in Uganda needs to be further clarified.

## Background

*Helicobacter pylori *is one of the most common causes of bacterial infection in human beings [1], and was first isolated and cultured by Warren and Marshall in 1983 [2]. It is a Gram-negative bacterium that inhabits the mucous layer of the gastric mucosa of the human stomach. It can cause chronic gastritis and is associated with recurrent peptic ulcer and gastric cancer [3,4].

*Helicobacter pylori *colonization is thought to be acquired early in life. Different theories of how *Helicobacter pylori *is acquired have been published, but no certain environmental source has been identified [5]. In fact it has been proposed that humans are the only reservoir for *Helicobacter pylori *[6]. Early colonization in children living under poor socio-economic conditions has been demonstrated, and several studies have shown a high prevalence of *Helicobacter pylori *among people in low-income countries [7-10]. Many studies from sub-Saharan Africa have been performed using serological tests [11-13], or the studies have been carried out in rural areas [12,14,15].

13 Urea breath test [16,17] or invasive methods, such as gastroscopy with biopsies and/or urease tests used to be the "gold standard" for detection of Helicobacter pylori. These are technically advanced, time consuming methods and unsuitable for children. Serological tests are also available but in children they often show lower specificity [18-20]. A major drawback of serological tests is that it does not discriminate between current and past infections. The faecal monoclonal antigen test has a high sensitivity, specificity and accuracy in children, 91-96%, 95-96% and 94-96% respectively [21,22]. The faecal test can be performed on humans in all age groups and gives a rapid result without the need for sophisticated laboratory equipment. Hitherto, no such studies have been carried out in children in Uganda.

The objective of this study was to determine the prevalence of *Helicobacter pylori *colonization in apparently healthy children aged 0-12 years in urban Kampala, Uganda.

## Methods

### Study design

This was a cross-sectional survey of *Helicobacter pylori *colonization in apparently healthy children aged 0-12 years in one part of urban Kampala, Uganda.

### Study site

**Figure 1:**
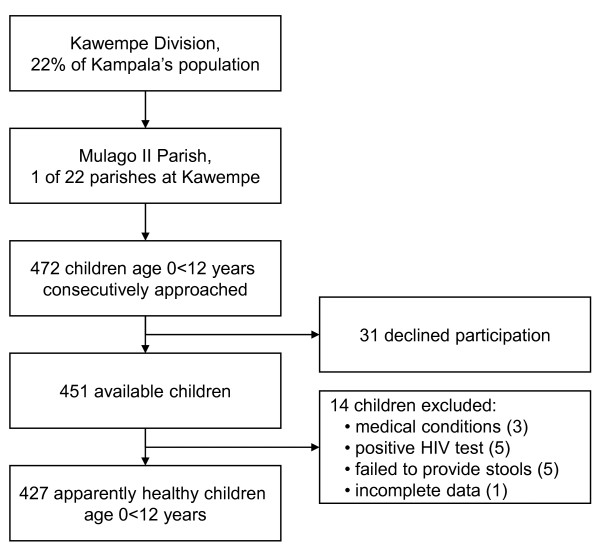
Study profile

The study was conducted in October-November 2007 in Kampala, the capital of Uganda with an estimated population in 2008 of 1.5 million inhabitants. Kampala has five divisions, one of them being Kawempe. Kawempe division houses 22% of Kampala's population. The study was carried out in all zones of Mulago II parish, one of the 22 parishes of Kawempe division, figure 1. Children less than 12 years comprise 35.3% of the population [23,24]. We purposely selected this resource limited area of the town, characterized by informal settlements, congested living, lack of proper sanitation conditions and low education level among adults. Plan International^® ^has provided the parish with tap water at subsidized prices.

### Study population

In the Mulago II parish children aged 0-12 years were recruited consecutively by door-to-door visits, an equal number of children in each age category of 0- < year, 1- < 3 years, 3- < 6 years, 6- < 9 years and 9- < 12 years (around 85 per age group), table 1. Participants were included in the study if: 1) they were apparently healthy, 2) aged between 0- < 12 years, 3) had an informed consent from caretaker and 4) were able to produce a stool sample within three consecutive days. All participants and their primary caretaker were offered a voluntary HIV test with pre- and post-test counselling, following the Ugandan national guidelines [25]. Of the 472 children approached, 31 declined participation (6.6%). Fourteen potential participants (3.1%) were excluded into the final analysis due to medical conditions (3), positive HIV test (5), failure to produce a stool sample in 3 days (5), incomplete data (1), figure 1. Children reporting chronic cough/asthma were included as other studies have not shown a significant correlation between *Helicobacter pylori *colonization and asthma [26].

**Table 1:** *Helicobacter pylori *colonization by age group

Age categories	Total number	***H. pylori *positiv*e***	***H. pylori *prevalence**
	N	n	% (95%CI)
0 < 6 months	39	13	33.3 (18-49)
6 < 12 months	48	12	25.0 (12-38)
1 < 3 years	87	40	46.0 (35-57)
3 < 6 years	89	46	51.7 (41-62)
6 < 9 years	84	46	54.8 (44-66)
9 < 12 years	80	32	40.0 (29-51)

Total	427	189	44.3 (40-49)

N/n = number

### Data collection

For the data collection, we recruited six Ugandan nurses with prior experience in data collection and who had experience in HIV pre- and post-test counselling; and two laboratory technicians. EH and GN trained them in stool sampling, interview technique and ethical issues. A pilot survey was conducted in an adjacent parish prior to the study, and the experience gained was used to adapt the procedures and the questionnaire in the final survey. The data collectors worked in teams of two persons. The survey was conducted with the support of the local official who introduced the survey team to the caretakers. The principal investigator followed the research assistants to the field daily, and if not present could be reached by mobile telephone and was able to reach the field within 30 minutes. Recruitment of children was done consecutively by door-to-door visits over a period of four weeks. We used guides with knowledge of the community to identify households with children in our target age. The guides also helped in follow up within the following 3 days and collecting stool samples from those children who were unable to produce stool on the survey date. The number of children included in each zone depended on the population density in each of the seven zones of the parish. A questionnaire was used to collect information on demographic data, family structure, socio-economic status of the family and health status of the participating child, in order to control for potential bias.

### The Helicobacter pylori stool antigen test

A stool sample was requested from each participating child and was collected in air tight containers either at time of the encounter, at the end of the day, or the following morning. Stool samples were transported from the field to the laboratory at ambient temperature twice daily and stored in a + 4°C fridge until the same afternoon or the following day when analysis were carried out. The *Helicobacter pylori *stool antigen test, HpSA^®^ImmunoCardSTAT was used to analyze the stool samples. It is a rapid lateral flow immunoassay that utilizes a monoclonal anti-*Helicobacter pylori *antibody as the capture and detector antibody. Instructions given by the manufacturer were followed. After every 20 tests the provided positive control test was performed. All positive control tests tested positive. Approximately 100 μl of stool was brought into the sample diluent vial and vortexed for fifteen seconds. Four drops of the specimen were applied to the test and the result was read after five minutes. The results were reported as positive or negative on the basis of the manufacturer's cut-off values.

### HIV-testing

In order to assess the *Helicobacter pylori *prevalence among healthy, non-HIV-infected, children in this high endemic area, all participants and their caretakers were offered a voluntary HIV test. Pre- and post-test counselling was provided according to Uganda guidelines [25]. It was performed after the interview on a finger prick blood sample. The screening test used was Determine HIV1/2^® ^(Abbott). If a child or a caretaker was considered HIV positive, they were referred to Mulago hospital, either Paediatric Infectious Disease Clinic (PIDC) or adult Infectious Disease Clinic (IDC) for follow up. All investigations and treatment at PIDC and IDC were free of charge.

### Sample size

We used OpenEpi http://www.openepi.com to calculate the sample size. The assumptions included 50% prevalence of *Helicobacter pylori *colonization and a 95% CI for the estimates:

Sample size n = [DEFF*Np(1-p)]/[(d2/Z21-α/2*(N-1)+p*(1-p)]. This gave us a sample size of 384 children. We added another 10% to allow for contingency, a total of 422.

### Statistical analysis

Data from the questionnaires and the results of the HpSA test were doubly entered using EpiData version 3.1 http://www.epidata.dk. The data were exported to SPSS version 15.0 for statistical analysis. Data quality was ensured through careful selection and training of research assistants, supervision, field editing by use of the "check" module at data entry combined with double data entry and validation. To explore the prevalence of *Helicobacter pylori *and its association to other factors, binary logistic regression as well as multiple logistic regression were performed. In the multiple logistic regression analysis adjustments were made for age, sex, type of housing, number of people in the same household, education level of the mother/female caretaker, drugs taken in the last tree months, toilet type (pit latrine), sharing the toilet with other families, sources of drinking water and wealth index. The confidence interval (CI) reported was set to 95%, the significance level was set to 0.05.

To explore the socio-economic status of the participants, principal component analysis (PCA) was used [27,28] based on 13 questions capturing socio-economic status, composed of assets in the household, sources of water and power available for the family and standard of housing for the child. The Kaiser-Meyer-Olkin value, shoving the strength of connection between variables, was 0.82, exceeding the recommended value of 0.6, and the Barletts Test of Sphericity reached statistical significance. The PCA revealed the presence of three components with Eigen values exceeding 1. The first principal component explained 31.3% the variance. The first principal component was chosen as our wealth index. The wealth index was ranked and categorized into 3 tertiles (1 poorest, 3 less poor). The tertiles were equally distributed.

### Ethics

Ethical approval was obtained from Makerere University, Faculty of Medicine, Research and Ethics Committee in Uganda and the Regional Committee for Medical and Health Research Ethics, West-Norway (REK-VEST) in Norway. The data collectors were trained in ethical issues prior to the study. Oral and written information about the study was given to the caretakers either in English or the local language. Informed consent was obtained from all the caretaker of the participants in the study.

## Results

The mean age (± SD) of the participants was 4.8 (3.6) years, for girls 5.2 (3.7) years and boys 4.3 (3.5) years. The youngest enrolled child was 5 days; there were 5 children younger than one month enrolled. The genders were equally represented in the study, 216 (50.6%) girls and 211 (49.4%) boys.

**Figure 2:**
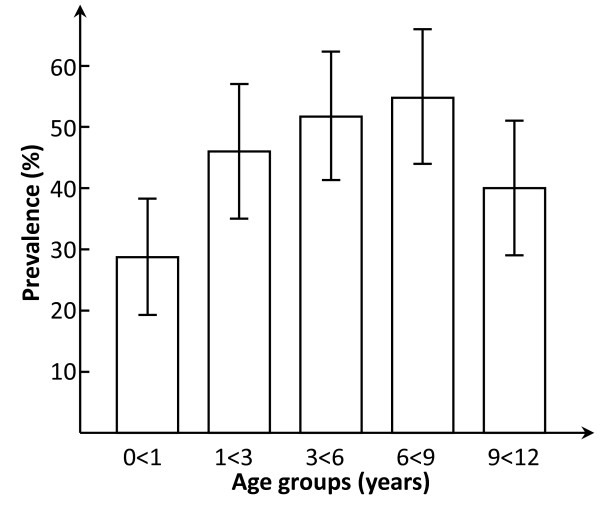
Prevalence (95% Confidence intervals) of *Helicobacter pylori *by age group

The overall prevalence of *Helicobacter pylori *colonization in the 427 children was 44.3%, table 1. Early colonization was common with a prevalence of 28.7% in children younger than 1 year of age, (33.3% below 6 months and 25.0% 6 < 12 months). There was a steady increase with increasing age (1- < 3 years 46.0%, 3- < 6 years 51.7%, 6- < 9 years 54.8%), figure 2. The prevalence in the oldest age group 9- < 12 years was somewhat lower than the previous age groups (40.0%), but the difference was not significant. The difference between the two lowest age groups (0- < 1 year and 1- < 3 years) was significant with an odds ratio and 95% confidence interval (OR ± 95% CI) of 2.3 (1.2-4.3). Out of the 189 *Helicobacter pylori *positive children, 84 (44.4%) were girls and 105 (55.6%) boys. The difference was significant, also after adjusting for socio-economic factors (table 2).

**Table 2:** *Helicobacter pylori *colonization and associated factors

	Number	HP positive	Unadjusted OR	**Adjusted**^**1 **^**OR**	**p-value**^**2**^
	N	n (%)	(95%-CI)	(95%-CI)	
**Age groups**					
0 - < 1 year	87	25 (28.7)	1	1	
1 - < 12 years	340	164 (48.2)	2.3 (1.4-3. 9)*	2.4 (1.4-4.1) *	0.002
					
**Sex**					
Female	216	84 (38.9)	1	1	
Male	211	105 (49.8)	1.6 (1.1-2.3)*	1.6 (1.1-2.4) *	0.02
					
**Type of housing**					
Permanent house	182	70 (38.5)	1	1	
Semi-permanent house	245	119 (48.6)	1.5 (1.0-2.2) *	1.8 (1.2-2-7) *	0.02
					
**Number of people in same household**					
2 - 4	143	55 (38.5)	1		
≥ 5	284	134 (47.2)	1.4 (0.95-2.2)	-	0.39
					
**Education level of mother/female caretaker**					
Completed secondary school or higher level	84	32 (38.1)	1		
Not completed secondary school	343	157 (45.8)	1.4 (0.8-2.2)	-	0.32
					
**Drinking water**					
Unprotected sources	43	17 (39.5)	1		
Public tap	384	172 (44.8)	1.24 (0.7-2.4)	-	0.67
					
**Using pit latrine**					
Yes	412	182 (44.2)	1		
No	15	7 (46.7)	1.1 (0.4-3.1)	-	0.94
					
**Sharing toilet with other families**					
Yes	401	170 (42.4)	1	1	
No	26	19 (73.1)	3.7 (1.5-9.0)*	3.7 (1.5-9.3) *	0.006
					
**Wealth index**					
Poor	142	61 (43.0)	1		
Poorer	142	60 (42.0)	1.2 (0.8-1.9)	-	0.64
Poorest	143	68 (47.9)	0.96 (0.6-1.5)		0.55
					
**Taken drugs last 3 months**					
Yes	345	149 (43.2)	1		
No	82	40 (48.8)	1.3 (0.8-2.1)	-	0.40
					
**Taken any antibiotics last 3 months**					
Yes	166	65 (39.2)	1		
No	261	124 (47.5)	1.4 (0.9-2.1)	-	0.38
					
**Reporting abdominal pain more than 3 times/week**					
No	400	176 (44.0)	1		
Yes	27	13 (48.1)	1.2 (0.5-2.6)	-	0.58

* p-value < 0.05

^1 ^adjusted for age, sex, type of housing, number of people in the same household, education level of the mother/female caretaker, if drugs taken the last tree months, using a pit, latrine, sharing the toilet with other families, sources of drinking water and wealth index

^2 ^p-value after adjusting for^1^

N/n number

OR Odds ratio

CI Confidence Interval

HP *Helicobacter pylori*

Children living in semi-permanent houses had a higher prevalence of *Helicobacter pylori*, 48.6% as compared to children in permanent houses, 38.5%. This difference remained significant after adjustment, table 2.

If the child household consisted out of 5 or more persons, there was a trend of larger risk of being colonized with *Helicobacter pylori *compared to households with 2-4 persons, but after adjustment this difference was insignificant, table 2. Children with a well-educated female caretaker (completed secondary school) compared with the others, showed no significant difference in *Helicobacter pylori *prevalence after adjustment.

For 89.9% of the children, the main source of drinking water was from a public tap or tap water into house/plot. Only 10.1% of children were getting their drinking water from unprotected water sources like pond, river, spring, well and borehole. After adjustment there was no significant difference of the infection rate in the two groups.

Pit latrines were used by 96.5% of the families of the children. Use of a pit latrine or a flush toilet did not affect the prevalence of *Helicobacter pylori*. Of the families, 93.9% were sharing the toilet with other families. Not 'sharing toilet' with other families remained a risk factor for *Helicobacter pylori *colonization, also after adjustment, table 2. Excluding 'sharing toilet' from the adjusted model, table 2, only marginally altered the OR estimates of the other three variables in the adjusted model and none of the other variables were retained.

There was no difference in the prevalence of *Helicobacter pylori *between the poor, the poorer and the poorest groups of the society, using our wealth index. Hundred and sixty-six out of the three hundred and forty-five children who had used drugs last 3 months had used different kinds of antibiotics. There was no significant different in the prevalence of Helicobacter

pylori neither in the drug using group nor the antibiotic using group. There were no significant differences in prevalence by breastfeeding duration (shorter or longer than 24 weeks) or provision of vitamin A within the last 6 months. We could not find a statistical difference in reported abdominal pain by the caretaker in children colonized by *Helicobacter pylori *compared to those not colonized, table 2.

In the adjusted analysis, there were four factors that remained significant: age, sex, type of housing and not sharing toilet with other families, table 2.

Out of the 427 participants 13 reported to have abdominal pain more than 3 times per week and tested positive for *Helicobacter pylori*. These children were called to the community centre and a closer medical history was obtained and clinical investigations were performed by a doctor. If the children's symptoms were suspect of *Helicobacter pylori *infection the children were given a triple treatment of amoxicillin/claritromycin/omeprazole for 1 week. Twelve children, with a mean age (±SD) of 6.6 (3.3) years, received treatment. After providing stool sample, each participating child was offered mebendazole for treatment of intestinal worms if not received otherwise within the last 6 months.

## Discussion

This is the first survey describing the prevalence of *Helicobacter pylori *colonization among apparently healthy children in an urban area in Uganda. The study revealed a high overall prevalence rate of *Helicobacter pylori*, 44.3% and early colonization was common. Boys were significantly more often colonized than girls and those living in semi-permanent houses more often than those living in permanent houses.

The strengths of our study are a) a test method with high sensitivity, specificity and accuracy in comparison to other methods [21,22] b) the study population is representative for this community; c) few potential participants declined to participate (6.6%) and few potential participants were excluded, (3.1%). Only 5 children were excluded from the final analysis due to HIV infection and any selection bias is minimal. We made efforts to ensure that our study population was healthy, and therefore it can be used as reference population for future studies.

However, we have not controlled for *Helicobacter pylori *colonization in the caretakers or siblings. This could potentially bias our prevalence estimate upwards. High resolution molecular methods used recently in South-Africa [29,30] to resolve transmission of Helicobacter pylori are highly invasive which limits their feasibility in low-resource settings.

Our findings are comparable to findings from other sub-Saharan countries [5,31]. Very few studies carried out in sub-Saharan Africa have used the accurate, non invasive method as we did [32]. A study carried out in apparently healthy Kenyan children, using serological tests found a prevalence of 45.6% [10]. A study from Cameroon [7] using serological tests had results similar to ours with a prevalence of 37.5% in children younger than 3 years. We found an increasing prevalence with age. These findings are comparable with findings from Cameroon, Nigeria, Gambia and Egypt [7,9,13,33]. We detected a lower prevalence in children aged 9- < 12 years compared to 6- < 9 years. This finding is not comparable with, for instance, the prevalence found in the Ugandan study from Newton et al [34] in adults, where the prevalence of *Helicobacter pylori *in adults suffering from different kinds of cancer, except gastric cancer, was 87%. Our study population was distinctly different as all our participants were apparently healthy and children. A study from Iran also found a lower prevalence in children older than 14 years [35]. In the era before the stool antigen test became available, several studies also found a decrease in the prevalence with age [36,37], suggesting spontaneous eradication[38], better attention to health issues in older children, or use of antibiotics for other common diseases [36,39]. Another explanation of this finding could be an increasing antibody production with increasing age that may lead to the decline of the prevalence rate in older children [36,37]. Differences in types of *Helicobacter pylori *in adults compared to children, and differences in special gastric receptors have been suggested as other explanations for this decrease in prevalence [40]. Auto-curability among black children, age 7-21 years in USA was found to be 0.3% per year and 5.5% per year among white children in the same cohort [41]. Among Peruvian children, a spontaneous eradication of 7% per month was found (6-30 months old) [42]. In the youngest children age 0 < 6 months we found a high prevalence. A high prevalence has been found in neonates [43], decreasing in older babies and toddlers, suggesting an auto-curability and that acquisition of Helicobacter pylori infection in children does not necessarily result in persistent infection in all cases [44,45].

We found a significant gender difference in the prevalence of *Helicobacter pylori *colonization, boys being infected more often than girls. These findings can not be confounded by age differences between the sexes, as the boys were younger than the girls on average but had a higher overall prevalence of *Helicobacter pylori*. These findings are comparable with findings in adults and also a study in children from Cameroon [7] but no such gender difference could not be found in a meta-analysis of 10 studies conducted over the last 20 years [46].

Congested living with more than 4 people in the household was associated with an increase in *Helicobacter pylori *prevalence, and this is similar to findings in other studies [10].

In our study almost all of the participants used a pit latrine and shared toilet with other household. Not sharing the toilet with other families was a risk factor. The strong association with 'sharing toilet' could be a spurious association. Not including this variable in the analysis did not alter the adjusted model. We are uncertain about the interpretation of this association.

In our study the prevalence of *Helicobacter pylori *colonization was not increased with decreasing socio-economic status of the family as found in other studies from the same region [7,10,47]. A possible explanation is the small socio-economic differences in our study population as our study was conducted in one parish only and within an area characterized by informal settlements, congested living, lack of proper sanitation condition, low education level among adults and small variation in income per family.

Few of the *Helicobacter pylori *colonized children complained about abdominal pain and only 12 children, with a mean age (± SD) of 6.6 (3.3) years were treated with an eradication cure. However the role of *Helicobacter pylori *in children with recurrent abdominal pain is controversial [48-51]. This study did not support a relation between abdominal pain and colonization with *Helicobacter pylori*.

## Conclusion

The prevalence of *Helicobacter pylori *colonization among urban Ugandan children is high at an early age and increases with age. High prevalence is associated with age, gender and type of housing. The impact of *Helicobacter pylori *on children's health in Uganda needs to be further clarified.

## Competing interests

The authors declare that they have no competing interests.

## Authors' contributions

EH participated in the conception, design and implementation of the study, statistical analysis, interpretation and writing the manuscript. TT participated in the conception and design of the study, statistical analysis, interpretation and writing the manuscript. DKM participated in implementation of the study and performed the HpSA tests. GN participated in design and implementation of the study. LG participated in design of the study, interpretation and writing the manuscript. EO participated in the conception and design of the study, statistical analysis, interpretation and writing the manuscript. JKT participated in conception, design and implementation of the study. All authors read and approved the final manuscript.

## Pre-publication history

The pre-publication history for this paper can be accessed here:

http://www.biomedcentral.com/1471-230X/10/62/prepub
